# The Use of Leisure1Read before the Ontario Veterinary Medica Society, January 19, 1898.

**Published:** 1898-10

**Authors:** William M. Wilson

**Affiliations:** Hartstown, Pa.


### THE USE OF LEISURE.[1]

By William M. Wilson, V.S.,
HARTSTOWN, PA.

The subject which I have chosen for my reading this evening is one that, I think, should be carefully considered by the young veterinary surgeon starting in business, especially if he expects to build up a good practice and hold it.

The use of opportunities which are coming to us in the intervals of our future practice is the subject to which I would direct your attention.

The question how those intervals of leisure, the mornings and evenings, holidays and Sundays, and other leisure hours, should be spent, is one of the most important that can engage our attention.

Show me how a young man spends his leisure, and I will undertake to calculate pretty nearly what will be his station in life a few years hence. Show me how a young man spends his leisure, and

[1] Read before the Ontario Veterinary Medica Society, January 19, 1898.

I will show you what sort of character, what sort of success, he is likely to have in business life.

There are two principles that young business men ought to hold themselves to steadfastly, carefully, and intelligently. The first principle is this: Nothing can stand against willingness to work, to do your best regardless of a little extra time and labor. The young man who can steadily, thoroughly, and regularly attend to his business, and be content with doing his best, is going to the front regardless of difficulties. The other principle is that leisure moments can be bound up in the condition of great advance. These leisure hours are to be the test-stones by which your quality and your progress and your success, in the highest meaning of the term success, is to be tested.

You want to be efficient business men; you want to be resourceful, well furnished all around; and if it may be your desire to be distinguished in your community, you should make your business prosperous, so prosperous that it will help to make you efficient men, worthy of help in the community in which you live.

The first use of leisure for a young veterinary surgeon starting in business, if he is called to treat a patient and his time is not limited, which it is not likely to be at the start, is to be willing to stay a little longer and watch and study the symptoms, note the action of your medicine; don't be afraid to throw off your coat and do a little work, and show, by your willingness to do all in your power for the animal, that you are as much interested in the case as the owner. Show by your willingness to work and stay with the animal that you are willing to more than fill the prescribed measure of your service, if you wish to go to the front.

For the purpose and by the means of a large helpfulness, make yourself necessary to the community in which you live; it is for your interest to do this in order that you may succeed. The man who is accommodating, who is always ready to take a few extra steps, or do a little more work than the occasion demands, is the one who will not get the go-by to try some newcomer.

Do something to help you toward a larger education, better thoughts, and more adequate resources; something that will help you to understand more about your business in which you are engaged. There is an old Oriental proverb that says, "O, square thyself for use; a stone that will fit in the wall is not left in the way." So it is with the veterinary surgeon; if he squares himself for usefulness in the world he will not be found wanting employment.

Some one said to Daniel Webster when he commenced to study law, "There are too many lawyers now." His answer was, "There is always room at the top." So it is in all kinds of business. Some say there are too many veterinary surgeons, but I say every good man makes as much room as he can occupy.

But, after all, you are going to be something more than business men; you are going to be members of society, to be citizens. A good business man is almost by that fact a good citizen. One great question is, "What is a young man going to be worth to himself?" He cannot build a structure any larger than he puts in the foundation. Put in a foundation as large as possible of information, of established knowledge. Make yourself ready to fill any position in your line of profession. The young man who prefers to squander his time and strength in the saloon, or to stand on the street-corner smoking a charity cigar, waiting for something to turn up, or sit on dry-goods boxes whittling and finding fault with everything and everybody but himself, is by no means doing what he has a calling to do.

The young veterinary surgeon should keep his mind bright; he should learn to be orderly, to be cheerful, sociable, obliging, charitable, and kind; to have a plan in life, to use his leisure steadfastly in things that he believes to be most important; to give up the recreations that will interfere with culture, and dissipations that will prevent him from coming to the front. There is a proverb which says, "He that contemneth small things shall fall by little and little." I hold the contrary to be true, also; he who regards these small things shall rise by little and little, and come out to the front before and above all those who are bound simply to float on the current of existence and have what they call a good time.

---

The *Horseshoers' Journal* is publishing in current numbers replies from many prominent veterinarians on the use of oils, ointments, and various hoof-dressings.

Dried Milk.—According to a recent chemical analysis, this new food-product is found to be a yellowish, coarse powder, and it is claimed that it possesses all the nutrient qualities of fresh milk, and that one part of the dried product equals ten parts of fresh milk. Its composition is as follows: Total solid matter, 95 per cent.; albumin, 25 per cent.; fat, 24 to 25 per cent.; ash, 5 to 7 per cent.; milk-sugar, 40 per cent.

